# Design of Stripping Columns Applied to Drinking Water to Minimize Carcinogenic Risk from Trihalomethanes (THMs)

**DOI:** 10.3390/toxics6010018

**Published:** 2018-03-19

**Authors:** Joel Canosa, Vicenç Martí

**Affiliations:** Department of Chemical Engineering, Escola d’Enginyeria de Barcelona Est (EEBE), Universitat Politècnica de Catalunya (UPC), C/Eduard Maristany, 10-14, E-08019 Barcelona, Spain; joelcanosap@gmail.com

**Keywords:** stripping columns, drinking water, disinfection by-products, trihalomethanes (THMs), human health risk assessment, water technology design

## Abstract

The aim of this study is the application of a software tool to the design of stripping columns to calculate the removal of trihalomethanes (THMs) from drinking water. The tool also allows calculating the rough capital cost of the column and the decrease in carcinogenic risk indeces associated with the elimination of THMs and, thus, the investment to save a human life. The design of stripping columns includes the determination, among other factors, of the height (*H_OG_*), the theoretical number of plates (*N_OG_*), and the section (*S*) of the columns based on the study of pressure drop. These results have been compared with THM stripping literature values, showing that simulation is sufficiently conservative. Three case studies were chosen to apply the developed software. The first case study was representative of small-scale application to a community in Córdoba (Spain) where chloroform is predominant and has a low concentration. The second case study was of an intermediate scale in a region in Venezuela, and the third case study was representative of large-scale treatment of water in the Barcelona metropolitan region (Spain). Results showed that case studies with larger scale and higher initial risk offer the best capital investment to decrease the risk.

## 1. Introduction

Trihalomethanes (THMs) in drinking water are disinfection byproducts (DBPs) formed by the action of chlorine derivatives on precursors (organic matter, bromide) present in the final steps of drinking water treatment plants (DWTP) [1,2]. The most common THMs are chloroform or trichloromethane (CHCl_3_), bromodichloromethane (CHBrCl_2_), dibromochloromethane: (CHBr_2_Cl), bromoform, and tribromomethane (CHBr_3_) [3].

Drinking water that contains THMs could increase liver, kidney, or central nervous system problems, as well as risk of cancer. Weight-of-evidence for human carcinogenicity is B2 (probable human carcinogen) for CHCl_3_, CHBrCl_2_, and CHBr_3_ is C (possible human carcinogen) for CHBr_2_Cl [4]. In the European Union (EU) the Drinking Water Directive 98/83/EC concerns the quality of water for human consumption, and fixes a maximum concentration levels (MCLs) of THMs in drinking water of 100 µg·L^−1^. In the United States (US), the Environmental Protection Agency (USEPA) establishes also maximum concentration levels (MCLs) of 80 µg·L^−1^ THMs for drinking water [5].

MCLs regulatory values are protective values that are based on admissible chemical risk and consider the ingestion of water by an adult receptor as a main pathway. The use of drinking water in other scenarios, such as indoor swimming pools [6,7], involve other important significant exposure routes, such as dermal contact and inhalation of volatile THMs, which have to be included when dealing with the potential risk of these contaminants. The presence of more vulnerable receptors in the scenarios (e.g., child) and the revisions of toxicity values are additional reason to establish a risk-based methodology for each case study when THMs concentrations are close to MCLs.

Although drinking water production involves the formation of these unhealthy compounds, the biological risks of inadequate disinfection in the final steps of DWTP are far greater than the potential chemical risk of long-term exposure to THMs. Therefore, THMs are difficult to avoid due to the need for disinfectant excess to persist in drinking water.

The increase of concentration of THMs in drinking water depend on factors such as the use of chlorine as a disinfectant product and its concentration, increasing time from application to the water, high concentration of natural organic matter (mainly humic substances), higher water pH, high temperature, and high concentrations of bromide and ammonia in the raw water [1,2,3].

This knowledge about THM generation factors allows focusing the problem on the use of different disinfectants, the decreasing use of precursors (organic matter, bromide), and the treatment steps of THMs in the produced drinking water. Recent studies have reviewed the physical, physical-chemical, chemical, and biological treatment technologies for THM removal in drinking water, both applied to intermediate steps of DWTP and to the produced drinking water [1,8].

The treatment technologies to reduce THMs have to remove the contaminants at low cost and preserve the quality of the final drinking water. Physical treatments are a good alternative to achieve these goals without compromising the quality of water [1].

Stripping is a mature unit operation that removes THMs from water to air by using equipment that promotes an intimate contact of the phases. Packed columns in stripping have been extensively used in the treatment of wastewater with some soluble gases (e.g., NH_3_), as well as several volatile organic compounds (VOCs) in process industry [9]. Several combinations of stripping with other technologies as adsorption or the use of membranes allow the use of this technology within water treatment to eliminate or recover VOCs [10].

In the case of drinking water treatment, these technology has been used as an option to remove THMs once they are formed and, thus, at the final steps of DWTP. In this context, the starting situation is that chemical risk is much higher than biological risk and the goal of treatment methods has to decrease the concentration of THMs without compromising the biological risk. The optimal point of treatment of stripping would be as close as possible to the moment of use in order to ensure a proper disinfection, because stripping could also remove the excess of disinfectant.

By using counter-current stripping packed columns, chloroform has been removed successfully [3,11,12], but good contactor design (mass transfer values, air to water ratio) is critical for optimal performance. Among the advantages of the stripping technique applied to THMs are that it is directly focused on toxic volatiles (which are linked to an important pathway of the chemical risk) and that the technology can be easily combined with other physical treatments such as heating to improve THM volatility [8,13].

Total treatment cost of stripping packed column technology is a cost-competitive alternative compared with other technologies as aeration by diffused air, adsorption and clarification by using coagulants. In all cases, unitary cost of treatment decreases with increasing water treatment capacity and, thus, with population size. Capital costs of stripping columns is much higher that operating and maintenance cost, that are usually linked to the blowing of air and pumping of water [3,14].

## 2. Materials and Methods

The following methodology has been implemented in a software program run by MATLAB^®^ (The Mathworks Inc., Natick, MA, USA). Appendix A details the steps followed for the calculation of the parameters.

### 2.1. Driving Force and Double Film Theory

The design of the stripping column is based on the well-known concept of driving force [2,9,15]. Mass transfer of dissolved volatile organic compounds (VOCs) in the cross-section of any vertical point of 1-D stripping column is proportional to the difference between the concentrations of the VOCs in the water (the richest phase) and the concentrations of VOCs in the water equilibrated with the bulk air (the poorest phase). It involves a steady-state radial movement of VOCs from the richest phase to the poorest phase.

The double film theory (DFT) describes the mass transfer process of VOCs in air-water systems (heterogeneous systems) when the driving force occurs. In the bulk phases, far from the interphase, the mixture of the component is perfect. Once the component is closer to the inter-phase equilibrium, Henry’s law is accomplished. DFT applied to stripping assumes steady-state equilibrium and the absence of a chemical reaction.

The overall mass transfer coefficient expressed in air *K_G_* (mol∙m^−2^∙s^−1^·atm^−1^) could be obtained by using DFT from the air mass transfer coefficient *k_g_* (mol∙m^−2^∙s^−1^·atm^−1^), the water mass transfer coefficient *k_l_* (m∙s^−1^), and Henry’s law constant, *H* (atm·m^3^∙mol^−1^):

(1) $$\frac{1}{k_{g}}+\frac{H}{k_{l}}=\frac{1}{K_{G}}$$

The mass transfer coefficient *k_g_* and *k_l_* for packed stripping columns can be obtained from Onda correlations [9,16,17] and *H* (see Appendix A).

### 2.2. Equilibrium and Operation Lines

Equilibrium line is a representation of the equilibrium between the concentrations of water of a contaminant *c^i^* (mol∙m^−3^) versus gas partial pressure *p^i^* (atm) in the interface. A linear relationship is obtained in the case of diluted gases:

(2) $$p^{i}=H\cdot c^{i}$$

Operation line (OL) is a representation of the global column mass balance in a liquid concentration versus gas partial pressure graphic [9]. Assuming that there is not a longitudinal mixture of unwanted components between flows, and that there is clean air in the bottom at ambient constant conditions (1 atm, 20 °C) and diluted dissolutions (<1%), OL is simplified with a straight line relating the top and the bottom points:

(3) $$P^{top}=\frac{\left(C^{top}-C^{bottom}\right)\cdot H}{S^{*}}$$

where S* is the stripping factor, given by:

(4) $$S^{*}=\frac{H\cdot G\cdot \rho_{L}}{P_{T}\cdot L\cdot M_{L}}$$

where *G* and *L* are the molar flows (mol∙s^−1^) of pure water and air respectively, *M_L_* is the molecular weight of the water (Kg·mol^−1^), *ρ_L_* is the density of the water, and *P_T_* the total atmospheric pressure (atm).

### 2.3. Section Calculation

In order to specify the size of the section *S* (m^2^), it is necessary to know other features of the column:

(5) $$S=G_{w}/\left(\rho_{G}\cdot v_{G}\right)$$

This Equation is complicated to solve because it has to be indirectly calculated from the gas flow rate *G_w_* (kg·s^−1^), the air density *ρ_G_* (kg·m^−3^), and the gas velocity *v_G_* (m/s). This last term depends on the filling features and must be around 70% of the gas velocity which causes flooding ([16]) to ensure safe operation.

### 2.4. Height Calculation

In order to calculate the height of the column, *H_column_* (m), the overall gas height of the plate *H_OG_* (m) and the theoretical overall gas number of plates *No_G_* have to be considered [9,16]. Equation (7) shows the calculation of *H_OG_*, where *a* (m^−1^) is the effective interfacial contact area by volume of the fillings:

(6) $$H_{column}=H_{OG}\cdot N_{OG}$$

(7) HOG=GS(a.KG)·pT

Colburn’s equation [9] from the integral equation definition for dilute concentrations allows the calculation of the number of plates, *N_OG_*:

(8) $$N_{OG}=\int_{P^{bottom}}^{P^{top}}\frac{dp}{\left(P^{eq}-P\right)}\;=\frac{1}{1-S^{*}}Ln〚\left(1-S^{*}\right)\cdot \frac{H\cdot C^{top}}{H\cdot C^{top}-p^{top}}+S^{*}〛$$

### 2.5. Cost Estimation

For the preliminary design of a stripping column, a rough approximation is enough to obtain a basic capital cost estimation with a possible variation of 30% [16]. By applying the well-known six-tenths rule [18] as a scale factor to water treatment capacity flow, *q* (L·min^−1^) and taking information from historical data about costs (*DC_ref_*) for known treatment capacity (*q_ref_*) of similar projects [14] direct costs (*DC*) of stripping columns were obtained:

(9) $$DC=DC_{ref}\cdot n\cdot {\left(\frac{q/n}{q_{ref}}\right)}^{0.6}$$

where *n* is the number of stripping columns used to cover the treatment capacity.

DC costs consider the treatment unit, pipes, valves, electrical connections installation cost. DC costs include only capital costs and not cover the Operation and Maintenance costs, which could strongly depend on the elimination of THMs, ratio air to water and local costs [3]. It means that the DC costs are focused as initial installation costs.

The indirect cost (IC) was also considered and was 40% of *DC*, covering design, engineering, contractor’s fees and contingency [16].

Total fixed capital cost (FC) is defined as the sum of *DC* and IC costs.

The working capital cost (WC) was 5% of FC and includes the additional investment needed to start the column up and operate it to the desired conditions

The software developed a calculation of the costs of total investment (TC) as FC plus WC. These relationship implies that total investment cost is 1.47 times the direct costs. It has been assumed that the amortization period of the stripping column is 29 years, the same that exposure duration time (see *ED*). 

### 2.6. Carcinogenic Human Health Risk Assessment (HHRA)

Risk assessment of the individual THMs in drinking water was performed using classical HHRA [19]. As a final output, a carcinogenic risk index linked to the potential carcinogenic risks for a specific scenario in the different study cases was derived.

The scenario considered is an adult that drinks the water and uses it in the shower. The pathways considered are, therefore, the ingestion of water, the dermal adsorption through skin during the shower, and the inhalation of vapors in the shower.

Exposure assessment is based on the calculation of a lifetime of daily intake, *THM_intake_*, for each of the three pathways and for each contaminant [19]:

(10) $$THM_{intake}=\frac{C_{w}\cdot EF\cdot ED\cdot f}{BW\cdot AT\cdot 365_{days/year}}$$

where *C_w_* is the annual average concentration of each THM in water (mg·L^−1^), *EF* is the exposure frequency to the contaminated media (day∙year^−1^), *ED* is the exposure duration (year), *BW* is the body weight of the receptor (Kg), and *AT* is the average life time (year).

The term *f* is the intake rate of water IR_water_ (L∙water∙day^−1^) for ingestion. In the case of dermal contact the f value is the product of permeability coefficient K_p_ (cm∙h^−1^) multiplied by exposure time ET (h) and multiplied by the skin surface in contact with water, SA (cm^2^). For inhalation f is the intake rate of air IR (m^3^ air day^−1^) multiplied by volatilization factor VF (L∙m^−3^) from water to air inhalation and multiplied for absorption efficiency, ABS.

Corresponding data used for these calculations are presented in Table 1 and Table 2.

The concentrations of THMs in air were calculated by using a water-air volatilization factor model developed in [24] and empirical values for chloroform [25].

The model considered is the volatilization from a droplet to an indoor scenario without renewal of air, known as E2A in reference [24]. The modelling of volatilization factors (VF) for contaminants as a function of the Henry constant for E2A showed that theoretical VF reach the highest constant value for contaminants with Henry constants lower than the values for THMs. This means that VFs for all the THMs in the present study were expected to be similar to maximum values.

Some published works with full-size experimental showers such as [25] operating at 26–29 °C showed empirical values of 3.5–18 L·m^−3^ for chloroform; thus, 18 L·m^−3^ has been chosen for all THMs as a conservative value in the present study. The carcinogenic effects of the individual THMs in terms of slope factors (SFs) are compiled in Table 2 from risk assessment information system (RAIS) [23].

Finally, the total cancer risk index is calculated by multiplying the estimated intake (*THM_intake_*) for individual THMs by the appropriate pathway SF (*THM_SF_*) and adding the three exposure pathways [19]:

(11) $$Risk_{total}=\underset{Pathways}{\sum }\underset{THM\;species}{\sum }THM_{intake}\cdot THM_{SF}$$

The value of *Risk_total_* is a quantitative carcinogenic risk index that yields the ratio of additional cancer occurrences per number of people in contact with the drinking water containing THMs.

Analyzing the results of these indexes at the inlet and outlet of the stripping column, the number of potential cancers that can be avoided by installing the stripping technology is determined by:

(12) $$Potential\;cancers\;avoided=\left(Risk_{initial}-Risk_{final}\right)\cdot Population$$

## 3. Case Studies

A summary of the three case studies basic inputs is detailed in Table 3. These case studies are detailed in references [23,26,27]

### 3.1. Hypothetical Neighbourhood in Córdoba, Spain (ES)

In this small-scale scenario, the option to install this equipment in a hypothetical neighborhood located close to Guadalmellato reservoir in Córdoba, Spain is analyzed [26].

According to this case study, seasonal THM concentrations in the range 0.1–22 µg·L^−1^ were determined using sensitive, accurate methods at seven different treatment points in the municipal DWTP of Córdoba and its distribution network collecting samples in the four seasons of the year. In general, the plant uses chlorine dioxide (ClO_2_) and chloramines (NH_2_Cl) as disinfectants, and its main scheme treats the raw water for the distribution network.

The aim is to check the viability of installing a single stripping column in order to improve the quality of the water for a small part of the population: a neighborhood. Then, assuming that almost all THM concentration is due to chloroform CHCl_3_ and studying the worst case from the spatial and seasonal variability, an initial value of 22 µg·L^−1^ was considered. This value was the average concentration in the summer season in distribution system of the exit of DWTP, which was the worst point in time and space. Temperature clearly affects the increasing of THMs as summer and spring showed the highest seasonal values. The point of treatment under these conditions is clearly after the distribution system and before the use of water. A value of 10 µg·L^−1^ was chosen as a target, because the risk calculated for the scenario was close to 10^−5^.

### 3.2. San Diego (Carabobo Region), Venezuela (VE)

The region of Carabobo is located in the central part of Venezuela; it has a population of almost 2 million inhabitants. The water distribution is done by two different pumped systems known as ‘Sistema Regional del Centro I’ (SRC-I) and ‘Sistema Regional del Centro II’ (SRC-II) [27].

The interesting point is that due to the high organic matter accumulated in parts of SRC-II, high doses of chlorine (up to 60 mg·L^−1^) are required to disinfect the water. According to [27], and probably as a consequence of direct long-term solar exposure and organic matter, the annual average concentration of THM is above USEPA limits in all the region with a range 80–110 µg·L^−1^ for chloroform. The idea of this case study was to consider an intermediate scale to take advantage of the positive facts to implement the stripping column technology in order to improve water health quality for a population.

The corresponding concentrations of individual THMs at SRC-II determined by [27] are shown in Table 3 and are typical values for annual average concentration. The final target of THM concentration was set to 21 µg·L^−1^ in order to be similar than case study 3. This target value allow an exploration of THMs treatment in an intermediate range of THMs with chloroform as a main contaminant as in case study 2.

### 3.3. Sant Joan Despí DWTP (Barcelona), Spain (ES)

Almost half of the drinking water supplied in the Barcelona Metropolitan Area (where 4.5 million of inhabitants live) comes from Sant Joan Despí DWTP, situated at the lower part of the Llobregat river. According to [23] its main water treatment line is based on pre-oxidation (ClO_2_), coagulation/flocculation, sand filtration, ozonation, activated carbon, and chlorination.

When the new EU law was applied (1 January 2009) a new membrane treatment line was installed and came into operation to reduce the total THM concentration levels. The new technology was placed in parallel with the conventional line specifically, after the sand bed filtration. At this point, the flow is divided and 50% is treated with the new process. The main step in the implementation was reverse osmosis (RO) which carries a concentration reduction of the DBP precursors (bromide, iodide, and organic matter). Finally, the water is re-mineralized (REM) and mixed with water from the conventional process. Comparing contaminant evolution before and after the implementation of the new membrane treatment line, a reduction of THMs was noted [23].

In this case study, the idea is to compare this new membrane treatment line with the retrospective possibility of installing a group of stripping columns at the end of the conventional process to treat all the water flow (330,000 L/min) in the same manner. Therefore, the input of the stripping column was set to THM average annual concentration, which were values of the outlet stream in 2008. As a target values the total THM concentrations of the outlet stream in 2012 was considered [23] (see Table 3).

As may be seen, brominated THMs have a higher concentration than chloroform and the scale of the treatment is large (a few millions of inhabitants). The starting level is similar to case study 2, but here chloroform is not predominant In this case study the idea is to obtain several stripping columns that could be placed at the end of distribution net and cover a wider zone and compare stripping technology with the membrane technology already installed. Despite the very large treatment capacity considered, the water flow treated by each column will be similar in order of magnitude to case study 2.

## 4. Results and Discussion

### 4.1. Parameters of the Stripping Columns

Table 4 shows the parameters of the stripping columns designed for the three case studies. Appendix A describes in detail the calculation steps for these parameters.

The three case studies showed increasing size of the column due to greater airflow and greater removal of THMs. In the third case study, the direct application of the water flow yielded a height value of 17.43 m and a diameter of 10.95 m, both of which are not mechanically feasible according to well-known rules of thumb (Internal diameter: 0.5–3 m) [16]. The design solution for the third case study consisted in dividing the treatment capacity flow into different parallel columns maintaining the height and a diameter equal to or less than 3 m. Splitting the total flow of 14 stripping columns in parallel several times was required.

The volumetric airflow (*G_V_*) was calculated using an intermediate S* value of 0.9 in Equation (4). The best filling type and values for parameter *a* were calculated for each design. Under these conditions, neither liquid nor gas phase mass transfer resistance was predominant.

*H_OG_* values proved to be similar in the three case studies (around 3 m) because *G* was balanced with *S* values (both increasing with the size). *N_OG_* showed increasing values due to the increasing removal of THMs and volatility need in each case study.

### 4.2. Comparison of Simulated Parameters with Stripping Columns in the Literature

Table 5 is a compilation of information from the literature focused on the removal of THMs using stripping packed columns [11,12,27,28,29,30]. Input date ranges from the references are detailed in the upper part of the table. The output date include *N_OG_*, calculated from Equations (3) and (8), the value of *H_OG_*, calculated from real height and *N_OG_* using Equation (6), and the real value of *a.K_G_* using Equation (7).

The comparison of simulated values (Table 4) with literature values (Table 5) allows us to validate the feasibility of the proposed design parameters of the column.

The ranges of concentrations in Table 5 covered all the case study simulation inputs, but removal of THMs in the references is greater than in the case studies.

Sarmiento [27] and McCarty [30] have a similar order of magnitude in section and *G_v_* values to case study 1. Sarmiento [27] has an extreme *G_v_*/*L_v_* ratio that decreases N_OG_ values and increases *H_OG_* greatly, but real *a.K_G_* values are higher than proposed in the case studies. McCarty [30] also shows *G_v_*/*L_v_* ratio higher than case study 1 which decreases *N_OG_* values and increases *H_OG_* and *a.K_G_* values.

Houel et al. [28] has a similar order of magnitude in section and *G_v_* values to case study 2. In this case, *N_OG_* is lower than the value of case study 2 and *a.K_G_* is much higher.

The rest of the studies ([11,12,29]) have a smaller scale than case studies proposed, but *H_OG_* or *a.K_G_* values showed a similar range.

As a conclusion, all the real *G_v_*/*L_v_* ratios and removal of THM in references are greater and the height columns are shorter than in the case studies. The combined effect decreases *N_OG_* and shows *a.K_G_* values in references that are higher than in the case studies. This situation shows that the columns from the references have superior performance than what is simulated in the case studies and it lends support to the idea that the simulation of case studies is sufficiently conservative to validate its feasibility.

### 4.3. Ratio Cost to Carcinogenic Risk

Table 6 shows the decrease in carcinogenic risk due to the stripping of THMs and the potential cancer deaths avoided by applying Equations (10)–(12) and population and water flow values from Table 3 and Table 4. Final risk index for carcinogenic effects exceeds 10^−5^ in all cases showing unacceptable risk, though the concentrations were bellow MCLs.

For *DC* calculation, values of *DC_ref_* and *q_ref_* have been set in the program according to the THMs removal and the treatment flow following reference [14]. As removal of THM was below 90%, when water flow was in the range 1250–12,500 L·min^−1^ a reference cost of 210,000 Eu for the treatment of 2271 L·min^−1^ was used and when water flow was higher than 12,500 L·min^−1^ a reference cost of 1,850,000 Eu and a value of 22,710 L·min^−1^ was used.

As may be seen, all parameters increase from case studies 1 to 3, with the exception of the ratio cost-life that decreases, showing minimum values for case study 3.

Case study 1 shows that, for small communities and small initial concentrations, the investment of stripping columns to reduce risk is not a good option.

Case studies 2 and 3 have similar concentrations of THMs around legislation limits (>80 µg·L^−1^), similar removal with different distribution of THMs. Both cases, with larger populations (>120,000 inhabitants) indicate the investment is a good option to reduce the THM risk with an initial investment around 19 Eu/inhabitant.

These results could be explained with a dimensional analysis assuming that water flow to treat is proportional to the population covered and that decrement of risk is proportional to decrement of THMs concentration.

Under this assumptions, DC has a dependence of *q*^0.6^ and *n*^0.4^ (Equation (9)) and saved lives have a direct dependence of *q*, due to population, and decrement of concentration (Equation (12)). The final dependence of the ratio is *(q*/*n)*^−0.4^ and decrement of concentration to power −1. The value *q*/*n* is the flow treated by each column and it means the greater is this value and the decrement of concentration, the lower will be this ratio. As decrement of concentration is proportional to initial concentration and THMs removal, it could be stated also that the greater is initial concentration and THMs removal, the lower will be this ratio.

These easy model explains why case study 3, with a *q*/*n* = 23571 L·min^−1^ has a better ratio than case study 2 (15,000 L·min^−1^), even the high DC value. As it has been mentioned, DC depends on *n*^0.4^, that means that in case study 3 with 14 units will increase almost three times the cost compared with case study 2.

## 5. Conclusions

The first conclusion is the technical viability of the application of stripping columns for the treatment of drinking water in order to reduce its associated carcinogenic risk.

By using a well-established theoretical approach, the main features of the stripping columns have been calculated for three case studies using a software tool, covering several ranges of THM composition, treatment capacity, and population.

The comparison of the simulated parameters with real values compiled in references where stripping of THMs was used showed that gas to liquid flows and the removal of THMs were higher in the real cases using shorter columns, which means that the simulated columns offer viable performance in reaching THM reduction.

A risk scenario involving ingestion and showering with drinking water was considered. A direct relation between the initial and final THM concentrations and carcinogenic risk index associated was observed, with values above 10^−5^ (1 death per 100,000 cases) in all case studies.

Case studies with high water flow treatment (or served population) and small number of units and high concentrations and elimination of THMs offer a good option to reduce THMs risk. Case studies 2 and 3 allow the preventing of 9.3 and 258.3 cancer deaths with estimated rough capital costs of 228 and 150 keu.life^−1^, respectively.

## Figures and Tables

**Table 1:** Input parameters to determine the exposure of *THM*s through the three pathways.

Parameter	Value	Reference
*EF*, Exposure frequency (day·year^−1^)	365	[20,21]
*ED*, Exposure duration (year)	29	[20]
*BW*, Body weight (kg)	60.55	[20]
*AT*, Average time (year)	70	[19,20,21]
IR_water_, Water ingestion rate (L·day^−1^)	2	[19,22]
SA, Skin surface area (cm^2^)	17,200	[20]
Skin in contact with water (%)	80	[20]
ET, Exposure time (h·day^−1^)	0.315	[20]
IR_air_, Inhalation rate (m^3^·h^−1^)	0.75	[20]
ABS, Absorption eff. in alveoli (%)	50	[20]

Table 2 compiles the specific properties of the contaminants for HHRA.

**Table 2:** Specific properties and SF values of THMs for HHRA [23].

	CHCl_3_	CHBrCl_2_	CHBr_2_Cl	CHBr_3_
Permeability coeff. (cm·h^−1^)	8.9·10^−3^	5.8·10^−3^	3.9·10^−3^	2.6·10^−3^
SF Oral (kg·day·mg^−1^)	3.10·10^−2^	6.20·10^−2^	8.40·10^−2^	7.90·10^−2^
SF Dermal (kg·day·mg^−1^)	3.10·10^−2^	6.20·10^−2^	8.40·10^−2^	7.90·10^−2^
SF Inhalation (kg·day·mg^−1^)	8.05·10^−2^	1.29·10^−1^	8.40·10^−2^	3.85·10^−2^

**Table 3:** General information of the case studies.

	Case Study 1	Case Study 2	Case Study 3
Reference	[26]	[27]	[23]
Location	Córdoba (ES)	San Diego (VE)	Sant Joan Despí (ES)
Treatment capacity, *q* (L·min^−1^)	1260	15,000	330,000
*Population* (inh.)	3000	120,000	2,250,000
$CHCl_{3}\;$(µg·L^−1^)	22.0	70.0	9.4
$CHCl_{2}Br\;$(µg·L^−1^)	-	10.0	18.7
$CHClBr_{2}$ (µg·L^−1^)	-	2.0	28.8
$CHBr_{3}\;$(µg·L^−1^)	-	-	39.6
Input Total (µg·L^−1^)	22.0	82.0	96.5
Output Total (µg·L^−1^)	10.0	21.0	21.7
% THMs Removed	54.6	74.4	77.6

**Table 4:** Parameters for stripping columns from case study simulations.

Outputs	Case 1	Case 2	Case 3
Number of columns, *n*	1	1	14
*H* (atm·m^3^·mol^−1^)	3.7 × 10^−3^	3.5 × 10^−3^	1.5 × 10^−3^
*G_V_*/*L_V_* (-)	5.9	6.2	14.7
Gas flow, *G_V_* (L·min^−1^)	7457	93,502	345,691
Filling type	Hiflow ring Plastic-3·1/2”	Hiflow ring Plastic-3·1/2”	Envipac ring Plastic-3·1/8”
*a* (m^−1^)	103	103	95
*k_l_* (m·s^−1^)	1.08 × 10^−3^	1.08 × 10^−3^	4.59 × 10^−4^
*a.K_G_* (mol·m^−3^·s^−1^·atm^−1^)	119	119	96
Section, *S* (m^2^)	0.20	2.35	6.72
Pressure drop (mm Hg·m^−1^)	25.5	25.5	4.0
*H_OG_* (m)	3.91	3.88	3.71
P top (atm)	3.95 × 10^−7^	1.88 × 10^−6^	5.92 × 10^−7^
*N_OG_*	1.42	3.59	4.70
*H_column_*(m)	5.55	13.95	17.43

**Table 5:** Parameters for stripping columns from references.

Reference	[27]	[11]	[12]	[28]	[29]	[30]
Input data						
Sample	CHCl_3_	CHCl_3_	CHCl_3_	THMs	THMs	THMs
Input total (µg·L^−1^)	536. 638	2000	50–300	29–40	15.8	165.5
% THMs removed	97.5 > 99.9	55–90	55–87	45–82	71–85	23–51
*H_column_* (m)	4	1.05	0.9	1. 2.1. 2.5	2.4	2.4
Section, *S* (m^2^)	0.270	0.066	0.049	0.018	2.000	0.300
*G_v_* (L min^−1^)	6700–9680	2376	40	nr	147,000	4200
*G_v_*/*L_v_* (-)	1800–2600	nr	13–40	10–40	22	38
Calculated output data						
*N_OG_*	1.4–2.2 × 10^−2^	nr	0.14–0.73	0.35–1.22	0.68	0.15
*H_OG_* (m)	185–296	nr	1.2–6.5	1.8–6.0	3.6	16.1
*a.K_G_*(mol·m^−3^·s^−1^·atm^−1^)	134–194	59–334 *	125–662	-	10,227	869

nr, non-reported, * reported values.

**Table 6:** Risk, capital investment, and cost to saved lives ratio.

Outputs	Case 1	Case 2	Case 3
Initial risk	2.60 × 10^−5^	1.04 × 10^−4^	1.48 × 10^−4^
Final risk	1.18 × 10^−5^	2.65 × 10^−5^	3.32 × 10^−5^
Cancer deaths avoided (lifes)	0.043	9.3	258.3
Capital investment, *DC* (Eu)	216,114	2,118,552	38,899,070
Ratio cost-life (Eu·life^−1^)	5,073,100	227,801	150,596

## Appendix A. Calculations with the Software Tool

The software was developed in a Matlab environment and has been validated through bibliographic references to ensure correct calculation of the equipment (mass transfer coefficients, height, pressure drop), as well as carcinogenic risk.

Throughout this study and in the software calculations, the following steps were taken:

Input water flow, initial concentrations of individual THMs, target concentration of total THMs, and type of filling (ceramic, metal, plastic)

Calculation of total molar concentration of THMS and weighted *M*, *H*, *D_g_* (denoted as *X_weightened_* in general terms) from the n THMs:

(A1) $$X_{weighted}=\frac{\sum_{i=1}^{n}C_{i}^{top}\cdot X^{i}}{\sum_{i=1}^{n}C_{i}^{top}}$$

Calculation of the gas flow with *S** = 0.9 from Equation (4) as an intermediate value of the stripping column.

Calculation of *p^top^* using Equation (3) and calculation of % THMs removed

Application of the method described in Coulson [16] to calculate the section, S, and pressure drop

Use of Onda correlations (Equations (A2)–(A4)) to obtain *k_l_*, *k_g_*, and *K_G_* (Equation (1)):

(A2) $$k_{l}\cdot {\left(\frac{\rho_{L}}{\mu_{L}\cdot g}\right)}^{1/3}=0,0051\cdot {\left(\frac{L^{*}}{a\cdot \mu_{L}}\right)}^{2/3}\cdot {\left(\frac{\mu_{L}}{\rho_{L}\cdot D_{L}}\right)}^{-1/2}\cdot {\left(a_{p}\cdot d_{N}\right)}^{0,4}$$

(A3) $$\left(\frac{k_{g}}{a_{p}\cdot D_{G}}\right)=5230\cdot {\left(\frac{G^{*}}{a_{p}\cdot \mu_{G}}\right)}^{0,7}\cdot {\left(\frac{\mu_{G}}{\rho_{G}\cdot D_{G}}\right)}^{1/3}\cdot {\left(a_{p}\cdot d_{N}\right)}^{-2}$$

(A4) $$\frac{a}{a_{p}}=1-exp\left[-1,45\cdot {\left(\frac{\sigma_{c}}{\sigma }\right)}^{3/4}\cdot {\left(\frac{L^{*}}{a_{p}\cdot \mu_{L}}\right)}^{0,1}\cdot {\left(\frac{a_{p}\cdot L^{*2}}{\rho_{L}^{2}\cdot g}\right)}^{-0,05}\cdot {\left(\frac{L^{*2}}{\rho_{L}\cdot a_{p}\cdot \sigma }\right)}^{0,2}\right]$$

where:

*ρ* = density, kg·m^−3^	*μ* = viscosity, Pa·s
g = gravity constant, m^2^·s^−1^	***L** = liquid flow, kg·m^−2^·s^−1^**
*D* = diffusion coefficient, m^2^·s^−1^	*a_p_* = dry packing specific area, m^−1^
*d_N_* = nominal packing diameter, m	*R* = gas constant, atm·m^3^·K^−1^·mol^−1^
*σ* = surface tension of water, N·m^−1^	*G** = gas flow, kg·m^−2^.s^−1^
***σ_c_* = critical surface tension of packing, N·m^−1^**	
Subscript L refers to water and G refers to air	

- Calculation of *H_OG_* (Equation (7))
- Calculation of *N_OG_* (Equation (8))
- Calculation of minimum thickness, number of distributors, and height (Equation (6))
- Graphics of the concentration profiles and equilibrium and operation lines
- Calculation of capital costs from liquid flow
- Calculation of carcinogenic risk from initial and final concentrations and exposure parameters assuming the same percentage of reduction (Equations (10)–(12))

For further details about the software used, please check [31].
